# Air pollution and defensive behavior: Evidence from transaction data in China

**DOI:** 10.1371/journal.pone.0307295

**Published:** 2024-11-07

**Authors:** Qingqing Yang, Xinping Dong

**Affiliations:** 1 School of Finance, Southwestern University of Finance and Economics, Chengdu, Sichuan, China; 2 School of Business, Ningbotech University, Ningbo, Zhejiang, China; Menzies School of Health Research: Charles Darwin University, AUSTRALIA

## Abstract

This study presents empirical research about the defensive behavior of air pollution, that is, health insurance purchases. Using transaction-level data from a large insurance company, covering more than half a million insurance contracts from nineteen cities in China from 2014 to 2018, we empirically imply that an increase of 10% in AQI leads to a 0.37% uptick in the number of daily sales of health insurance contracts by the company within the city. The effect is non-linear and is more pronounced when the AQI exceeds 200. Besides, the defensive cost for a one-unit increase in AQI accounts for around 1.70% of individual income annually. We demonstrate that the positive impact of air pollution on health insurance purchases is primarily driven by health management awareness and social interaction.

## 1. Introduction

Research shows the detrimental effect of air pollution on health. However, the severity of air pollution varies across countries. According to the 2022 WHO air quality database, more than 80% of cities in high-income countries meet the WHO’s Air Quality Guidelines for PM2.5. In sharp contrast, fewer than 1% of cities in low- and middle-income countries adhere to these air quality standards. The WHO (2017) [1] contends that over 88% of premature deaths worldwide due to air pollution occur in low- and middle-income nations, particularly in China and India [2].

Studies on air pollution have primarily emphasized health risk assessments [3–5], labor productivity [6, 7], and economic costs [8–10]. Studies also have provided compelling evidence that air pollution exerts a direct impact on our physical and psychological health [11–15]. Nevertheless, it is still necessary to explore the behavior and extent to which individuals in low- and middle-income countries resort to self-protection in response to air pollution.

As we know, insurance plays a pivotal role in fostering an individual’s sustainable health development [16, 17]. In this paper, we aim to analyze whether and to what extent individuals in low- and middle-income countries with high pollution transfer the risks of air pollution through health insurance. Empirically, using insurance transaction data from China, a developing country where the yearly PM2.5 concentration exceeds the latest WHO annual threshold by a magnitude of sevenfold, we examine the defensive behavior, explore the mechanisms, and evaluate the economic cost.

Our dataset comprises unique insurance transaction records encompassing over half a million insurance contracts across nineteen cities in China, spanning from 2014 to 2018. For the empirical test, the dependent variable is the logarithm of the daily number of insurance sales in the city. We quantify air pollution levels using the Air Quality Index (AQI) and employ the logarithm of the average AQI over a three-day window in the city as the independent variable. We find a positive correlation between air pollution and health insurance purchases. In particular, we imply that an increase of 10% in AQI results in a 0.37% uptick in the daily sales of health insurance contracts by the company within the city. Furthermore, the effect of pollution on the purchase for health insurance is non-linear and is more pronounced when the AQI exceeds 200. Compared to the demand for health insurance on “blue sky” days (AQI ≤ 100), purchases increase significantly by 4.43% on very unhealthy days (200 < AQI ≤ 300) and by 11.68% on hazardous days (AQI > 300).

We construct several additional tests to address the potential endogeneity concerns in our study. First, we employ the non-health insurance sold by the same company as the placebo test, consisting of accident and endowment insurance. The result shows no discernible impact of air pollution on the decision to purchase non-health insurance, mitigating any omitted variables bias stemming from the specific company characteristics. Second, we use thermal inversion and ventilation coefficient as instrumental variables for air pollution [18–21]. It is well known that ventilation coefficient and thermal inversion are exogenous meteorological phenomena. Our 2SLS estimations confirm a positive and statistically significant correlation between instrumented air pollution and health insurance purchases. Third, we conduct a difference-in-difference (DID) analysis using the “2+26” plan, that aims to reduce air pollution in Beijing, Tianjin, and 26 other cities located in the Beijing-Tianjin-Hebei area every year from 2017 to 2019. Since the plan does not directly impact the demand for health insurance but only reduces regional air pollution, the interpretation of DID estimations also implies a causal correlation between air pollution and insurance purchases.

To elucidate the mechanisms underlying the insurance effect associated with air pollution, we first find that the marginal effect of air pollution is more significant among individuals with greater awareness of health management. Another explanation for our findings is social interaction, aligning with Hu (2022) [22], which demonstrates that social interaction significantly contributes to the effect of insurance purchases through information transfer.

As the defensive cost cannot be directly predictive, we provide a perspective on the economic magnitude by estimating the marginal willingness to pay for air pollution [23]. Our results are similar with Gao et al. (2023) [24], showing that individual is willing to pay 302.23 Chinese Yuan per year to defend against a one-unit increase in AQI, accounting for approximately 1.70% of consumers’ income.

Our contribution to the literature is multifaceted. First, the paper enriches the studies on air pollution defensive behavior and defensive cost by exploring long-term risk management. Short-term physical defenses against air pollution have been analyzed from the perspective of air filters and anti-PM2.5 masks [23, 25]. We find that air pollution significantly promotes health insurance purchases, adding to the growing literature on defensive behavior in response to air pollution. Second, our results emphasize the importance of air pollution as an environmental factor in insurance decisions. Insofar influences of insurance demand are education, income level, financial literacy, and so on [26, 27]. The study finds that air pollution leads to the demand for insurance as well, which provides additional evidence of the environmental effects on finance-related behaviors. Third, we test the mechanisms of air pollution effects empirically from the perspectives of health management and social interaction. We propose that health management awareness and information access underlie the observed causal effect of air pollution and demand for health insurance.

The remainder of the paper proceeds as follows. Section 2 reviews related literature. Section 3 describes the data and introduces empirical models. Section 4 presents our estimation results for the causal effect of air pollution on defensive behavior. Section 5 explores mechanisms for our findings. Section 6 estimates the defensive marginal willingness to pay for air pollution. Section 7 concludes.

## 2. Related literature

Air pollution, referred to as an “invisible killer”, is accountable for profound adverse impacts on morbidity and mortality [3, 5, 9, 10, 28]. Thus, there is no doubt that air pollution induces avoidance and defensive behaviors.

In the first strand, the literature highlights households proactively select where to live. Bayer et al. (2009) [29] and Chay and Greenstone (2005) [30] provide evidence that US households vote with their feet to seek a living environment in response to air pollution. Similarly, air pollution is also responsible for settlement intention in China, either well-educated people or migrant workers [21]. Especially after real-time pollution information is publicly available, migration decisions due to air pollution become much more prominent [24]. Besides, people are more likely to migrate from polluted to cleaner cities, leading to a 43% reduction in their exposure to extreme pollution [31].

In the second strand, prior work also focuses on defensive investments. Liu et al. (2018) [32] assess the correlation between air pollution and online searches for air filters and anti-PM2.5 masks. Undoubtedly, a higher volume of online searches is followed by increased purchases. Utilizing unique data from online sales indices, Ito and Zhang (2020) [23], along with Zhang and Mu (2018) [25], demonstrate that individuals tend to purchase more air filters and anti-PM2.5 masks during periods of severe pollution.

Furthermore, insurance serves as an important channel for spreading and transferring risks [17]. Most studies analyze the individual insurance decisions affected by air pollution using household survey data [33, 34]. However, household survey data cannot pinpoint the exact timing of residents’ insurance purchases. Moreover, using annual average air pollution levels at the city or province to estimate the consequences further complicates the accuracy. Therefore, there is still a lack of evidence to study individuals’ insurance behavior and economic costs in response to air pollution based on accurate daily micro-transaction data. Chang et al. (2018) [35] study the relationship between air pollution and health insurance by employing data from an insurance company in a few Chinese cities. Our research mainly differs from Chang et al. (2018) [35] in five aspects. Firstly, the sample of Chang et al. (2018) [35] comes from a limited number (n < 5) of large cities in China. Our insurance data comprises nineteen cities in China, which is broader and more representative. Secondly, Chang et al. (2018) [35] estimate the impact of high hourly PM2.5 over a two-day window, while we pay attention to average AQI over a three-day period. For one thing, high hourly concentrations of PM2.5 are episodic and cannot fully represent ambient air pollution exposure. For another, the Chinese real-time pollution monitoring and disclosure program compiles the AQI, which is public and direct for people to access. Overall, our independent variable is more reflective of ambient air pollution exposure and public access to pollution information. Thirdly, we provide a cleaner identification of the effect of air pollution on health insurance purchases. We not only apply the placebo test and instrumental variable method to exclude endogeneity, but also construct DID analysis to confirm the causal effect. Specifically, we also cluster the standard errors to city-year level, which is more reliable than the robust standard errors of Chang et al. (2018) [35]. Fourthly, Chang et al. (2018) [35] propose that the demand for health insurance may be perceived as irrational when exposed to severe air pollution, particularly considering the cancellation effect during the cooling-off period. The paper empirically explores the mechanisms mainly associated with health management awareness and information access. Lastly, considering the defensive cost cannot be directly predictive, we further take into account the corresponding economic magnitude by estimating the marginal willingness to pay to defend against air pollution.

## 3. Data and empirical strategy

### 3.1 Data

#### 3.1.1 Insurance transaction-level data

Our transaction-level insurance data are sourced from nineteen Chinese cities, covering the period from 2014 to 2018, obtained from a prominent Chinese company. This company ranks among the first ten insurance firms established in China. Our data collect comprehensive details of more than half a million insurance contracts, including health insurance and some other products, each of which includes the city of purchaser, date of purchase, premium per annum, as well as some basic demographic information such as age and gender of the consumer.

#### 3.1.2 Air quality and meteorological data

To quantify air pollution, we utilize the Air Quality Index (AQI) provided by the Ministry of Environmental Protection of China, which is compiled by SO2, O3, CO, PM2.5, PM10, and NO2. The Chinese central government has pushed forward third-party environmental monitoring since February 2015 to address data manipulation issues and improve the accuracy of air quality [36]. Our research mainly focuses on the period later than 2015 to ensure the reality of air quality. We use the average AQI over a three-day window, considering the possible lagging effect in implementing purchase decisions.

The AQI is divided into six categories based on its various health effects: I-good (AQI ≤ 50), II-moderate (50<AQI ≤ 100), III-unhealthy for sensitive groups (100<AQI ≤ 150), IV-unhealthy (150 < AQI ≤ 200), V-very unhealthy (200 < AQI ≤ 300) and VI- hazardous (AQI > 300). An AQI below 100 is commonly referred as “blue sky” in China, indicating a suitable level of air quality for human health. Thus, we combine the first two categories and construct five indicator variables.

We collect daily weather information for the cities in our sample from the China Meteorological Data Service Centre, which includes data on temperature, humidity, wind speed, and precipitation.

#### 3.1.3 Ventilation coefficient

According to Hering and Poncet (2014) [18], the ventilation coefficient is calculated as the product of the boundary layer height and wind speed. We obtain the data from MERRA-2^1^, where the boundary layer height and hourly surface wind speed are available at a 0.5° * 0.625° (approximately 50 km × 60 km) latitude by longitude grid level. We first collect the ventilation coefficient in each grid for each hour, and then average the indicator to every day. Next, we match the grid to the city and average the indicator over a three-day window.

#### 3.1.4 Thermal inversion

The thermal inversion data is also from MERRA-2^2^, which reports the temperature of 42 atmospheric pressure layers every 6 hours for each 0.5° * 0.625° (around 50 km×60 km) latitude by longitude grid. To apply the data to the city, we match it from the grid and calculate the temperature difference between the second layers (975hpa, 320m) and the first layers (1000hpa, 110m) every 6 hours. A thermal inversion is defined to occur when the difference is positive. Next, we aggregate the number of thermal inversions occurring over a three-day window.

### 3.2 Empirical strategy

To examine our model prediction empirically, we employ the following specification model:

$$L\text{og}\_Num\_contract_{it}=\alpha_{1}Log\_L3\_AQI_{it}+\alpha_{2}X_{it}+D_{it}+\varepsilon_{it}$$ (1)

where *Log_Num_contract*_*it*_ is the natural logarithm of the number of health insurance contracts sold by the company in city *i* on date *t*. *Log_L3_AQI*_*it*_ is the natural logarithm of the average AQI of the city *i* over a three-day window considering the potential lagging effect in implementing purchase decisions, which consists of date *t*, *t-1*, and *t-2*. We employ a dynamic model with a range of lags to explore whether the three-day window sufficiently reflects the lagged effects [28]. As reported in Table 1, the largest response to pollution occurs on the simultaneous day, and the cumulative effects of air pollution increase steadily within three days, fluctuating subsequently. Specifically, we further test the differences in cumulative effects across different lags compared to the three-day window cumulative effect using the bootstrap testing method. The results show that the difference between the cumulative effects of the one-day and two-day windows compared to the three-day window is significant at the 5% level. In contrast, the difference between the cumulative effects of the four-day and longer windows compared to the three-day window is not statistically significant. Therefore, we believe that a three-day window would be appropriate for our study. Besides, we also estimate the impact of air pollution on health insurance using different window periods as robustness checks.

**Table 1:** Cumulative dynamic estimates of the effect of air pollution and health insurance purchases.

	Health Insurance						
Variables	(1)	(2)	(3)	(4)	(5)	(6)	(7)
*Log_AQI*_*t*_	0.0393***	0.0294***	0.0311***	0.0313***	0.0317***	0.0317***	0.0309***
	(4.092)	(2.831)	(2.981)	(3.009)	(3.046)	(3.080)	(2.999)
*Log_AQI*_*t-1*_		0.0187*	0.0092	0.0095	0.0103	0.0108	0.0111
		(1.861)	(0.749)	(0.763)	(0.811)	(0.853)	(0.879)
*Log_AQI*_*t-2*_			0.0149	0.0129	0.0123	0.0132	0.0135
			(1.376)	(1.068)	(0.992)	(1.065)	(1.085)
*Log_AQI*_*t-3*_				0.0034	0.0070	0.0057	0.0059
				(0.321)	(0.613)	(0.484)	(0.509)
*Log_AQI*_*t-4*_					-0.0046	0.0026	0.0031
					(-0.428)	(0.227)	(0.264)
*Log_AQI*_*t-5*_						-0.0102	-0.0107
						(-0.991)	(-0.986)
*Log_AQI*_*t-6*_							0.0019
							(0.179)
Cumulative Effect	0.0393***	0.0481***	0.0552***	0.05707***	0.0566**	0.0539**	0.0557**
	(4.09)	(4.33)	(4.61)	(4.02)	(3.55)	(3.01)	(2.82)
Diff with Cumu. Eff. (3)	-0.0159**	-0.0071**		0.0019	0.0014	-0.0013	0.0005
	(-2.45)	(-1.98)		(0.41)	(0.20)	(-0.16)	(0.05)
Adjusted *R*^*2*^	0.7142	0.7143	0.7142	0.7141	0.7142	0.7143	0.7143
N	34,656	34,637	34,618	34,599	34,580	34,561	34,542

Note: All regressions control for year-by-city, month-of-year-by-city, day-of-week, and holiday fixed effects, as well as a series of quadratic weather variables. The t-statistics are reported in parentheses based on standard errors clustered at the city-year level. Significance is indicated by

*** 1%

** 5%

* 10%.

Additionally, considering that the impact of air pollution on health is nonlinear, we explored whether there is a nonlinear relationship between air pollution and health insurance purchases. Thus, we replace the independent variables *Log_L3_AQI* with indicator variables corresponding to the AQI health effect categories.

*X*_*it*_ denotes control variables for weather conditions, comprising a quadratic function of the daily average temperature, cumulative precipitation, relative humidity, and average wind speed. The detailed definitions of control variables are introduced in S1 Table in the S1 Appendix. *D*_*it*_ is a battery of fixed effects, consisting of year-by-city, month-of-year-by-city, day-of-week, and holiday, to control the invariant confounding factors by city and by the specific cycle period (e.g., seasonal variation). Standard error terms are clustered at the city-year level.

### 3.3 Summary statistics

Our estimation sample consists of 34,656 city-date observations. Table 2 presents the summary statistics. The mean and standard deviation of *Num_contract* are 12.1913 and 23.587, respectively. The average *L3_AQI* is 90.108, with a standard deviation of 43.622. It also shows that approximately 69.5% of city-days are “blue sky”, and the remaining 30.5% are deemed unhealthy for humans. Concerning weather conditions, the average daily temperature is 17.136°C; the average cumulative precipitation is 3.669mm; the average relative humidity is 72.382%; and the average wind speed is 2.198m/s. Furthermore, the average *L3_VC* is 1586.030, and the average cumulative inversion times in a three-day window is 1.101.

**Table 2:** Summary statistics.

	Mean	S.D.	Min.	Med.	Max.	N
*Num_Contract*	12.1913	23.587	1	5	1319	34,656
*L3_AQI*	90.108	43.622	20	81	500	34,656
*I (AQI ≤100)*	0.695	0.460	0	0	1	34,656
*I (100 < AQI ≤150)*	0.211	0.408	0	0	1	34,656
*I (150 < AQI ≤200)*	0.057	0.232	0	0	1	34,656
*I (200 < AQI ≤300)*	0.030	0.170	0	0	1	34,656
*I (AQI >300)*	0.007	0.084	0	0	1	34,656
*Temperature (°C)*	17.136	9.663	-22.748	19.083	34.887	34,656
*Precipitation (mm)*	3.669	9.599	0	0.095	180.127	34,656
*Humidity (%)*	72.382	15.807	10.642	75.490	99.206	34,656
*Wind_speed (m/s)*	2.198	0.848	0.582	2.016	9.487	34,656
*L3_VC*	1586.030	622.386	418.797	1483.385	5635.630	34,656
*L3_Inversion_Num*	1.101	1.219	0	0.667	8	34,656

## 4. Results

### 4.1 Effects of pollution on defensive behavior

The results of OLS regression on the effects of air pollution are reported in Table 3. We begin with a model in column (1) that excludes control for weather variables. In column (2), we include extensive weather controls, as well as fixed effects of year-by-city, month-of-year-by-city, day-of-week, and holiday. Despite the decrease in the coefficient of *Log_L3_AQI* from 0.0488 to 0.0370, both results in columns (1) and (2) reveal a positive and statistically significant relationship between air pollution and health insurance purchases at well below the 1% level. An increase of 10% in AQI results in a 0.37% uptick in the daily sales of health insurance contracts by the company within the city.

**Table 3:** Effects of air pollution on health insurance purchases.

	Health Insurance			Other
Variables	(1)	(2)	(3)	(4)
*Log_L3_AQI*	0.0488***	0.0370***		0.0052
	(4.005)	(2.779)		(0.653)
*I (100 < AQI ≤150)*			0.0152	
			(1.625)	
*I (150 < AQI ≤200)*			-0.0065	
			(-0.392)	
*I (200 < AQI ≤300)*			0.0443*	
			(1.980)	
*I (AQI >300)*			0.1168**	
			(2.032)	
*Temperature*		-0.0018	-0.0012	0.0018
		(-0.653)	(-0.449)	(1.105)
*Temperature* ^*2*^		0.0003***	0.0003***	0.0000
		(3.158)	(3.223)	(0.432)
*Precipitation*		-0.0009	-0.0007	-0.0004
		(-1.152)	(-0.985)	(-0.725)
*Precipitation* ^*2*^		0.0000	0.0000	0.0000
		(0.268)	(0.157)	(0.265)
*Humidity*		-0.0026	-0.0024	0.0001
		(-1.115)	(-1.052)	(0.112)
*Humidity* ^*2*^		0.0000	0.0000	0.0000
		(1.278)	(1.118)	(0.119)
*Wind_speed*		0.0001	-0.0012	-0.0120
		(0.004)	(-0.068)	(-0.975)
*Wind_speed* ^*2*^		0.0008	0.0009	0.0006
		(0.276)	(0.300)	(0.309)
Year-by-City	Yes	Yes	Yes	Yes
Month-of-year-by-City	Yes	Yes	Yes	Yes
Day-of-week	Yes	Yes	Yes	Yes
Holiday	Yes	Yes	Yes	Yes
Adjusted *R*^*2*^	0.7142	0.7145	0.7145	0.5147
N	34,656	34,656	34,656	34,656

Note: The t-statistics are reported in parentheses based on standard errors clustered at the city-year level. Significance is indicated by

*** 1%

** 5%

* 10%.

It is possible that the effect of pollution on health insurance demand is nonlinear due to the non-linear health risks associated with AQI. Therefore, we re-estimate model (1) in column (3) with the indicator variables of AQI, as mentioned above, in place of the linear AQI variable. The AQI between 0 and 100, known as “blue sky”, which is typically considered good or moderate for human health, is the omitted indicator. The result indicates that the effect of pollution on health insurance purchases is pronounced when the AQI exceeds 200. Compared to the demand for health insurance on “blue sky” days, the purchase significantly increases by 4.43% on very unhealthy days (200 < AQI ≤ 300) and 11.68% on hazardous days (AQI > 300). These results further affirm the positive influence of air pollution on health insurance purchases.

### 4.2 Endogeneity

#### 4.2.1 Placebo test

Although we control for a range of fixed effects to address city and specific cycle period factors, one potential concern to consider is endogeneity pertaining to omitted variables, which may affect both air pollution and the demand for health insurance. For instance, air pollution is generally related to regional economic activities that can affect the demand for health insurance. Additionally, our results may be influenced by the characteristics of the insurance company since our health insurance transaction data is obtained from a specific provider.

Fortunately, we can weaken the concern by performing a placebo test with non-health insurance from the same company, which consists of accident and endowment insurance. We estimate the model (1) by replacing the dependent variable with the non-health insurance contracts. The result is presented in column (4) of Table 3, which shows that the impact of *Log_L3_AQI* is statistically insignificant and slight. These results suggest that air pollution is only significantly correlated with the demand for health insurance and has no business with non-health insurance. Overall, the placebo test mitigates the bias of omitted variables, such as the specific characteristics of the insurance company, to some extent.

#### 4.2.2 Instrumental variables for air pollution

Ventilation coefficient and thermal inversion are meteorological phenomena that are independent of any economic activities. The ventilation coefficient is expected to reflect a positive correlation with the dispersion speed of air pollution [18, 19]. Thermal inversion, in which air temperature increases with altitude, obstructs convection in the atmosphere and hinders the diffusion of pollutants [20, 21]. Therefore, we use the ventilation coefficient and thermal inversion as instrumental variables to alleviate endogeneity issues. The 2SLS models are specified as follows:

$$Log\_L3\_AQI_{it}=\theta_{1}Log\_L3\_VC_{it}/L3\_Inversion\_Num_{it}+\theta_{2}X_{it}+D_{it}+\varepsilon_{it}$$ (2)

$$Log_{-}Num_{-}contract_{it}=\vartheta_{1}Log\_\hat{L3_{-}A}QI_{it}+\vartheta_{2}X_{it}+D_{it}+\varepsilon_{it}$$ (3)

where *Log_L3_VC*_*it*_ is the natural logarithm of the average ventilation coefficient of city *i* over a three-day window and *L3_Inversion_Num*_*it*_ is the cumulative inversion times over a three-day period. $Log\_\hat{L3_{-}A}QI_{it}$is the instrumented value of *Log_L3_AQI*_*it*_ from Eq (2). We control for the same quadratic function of weather conditions *X*_*it*_ and fixed effects *D*_*it*_ used in the model (1). The 2SLS estimation results are reported in Table 4.

**Table 4:** Instrumental variables for air pollution.

	AQI	Health Insurance	AQI	Health Insurance
Variables	(1)	(2)	(3)	(4)
* $Log\_\hat{L3_{-}A}QI$ *		0.1161***		0.4898***
		(3.505)		(2.972)
*Log_L3_VC*	-0.6062***			
	(-23.388)			
*L3_Inversion_Num*			0.0414***	
			(8.041)	
Weather Controls	Yes	Yes	Yes	Yes
Fixed Effects	Yes	Yes	Yes	Yes
N	34656	34656	34656	34656
KP F-statistic	546.995		61.139	

Note: All regressions control for year-by-city, month-of-year-by-city, day-of-week, and holiday fixed effects, as well as a series of quadratic weather variables. The t-statistics are reported in parentheses based on standard errors clustered at the city-year level. Significance is indicated by

*** 1%

** 5%

* 10%.

The results of the instrumental variable *Log_L3_VC* are reported in columns (1) and (2), respectively. It is clearly that the coefficient on *Log_L3_VC* is -0.6062, significantly negative associated with air pollution. The Kleibergen-Paaprk F-statistic for the weak identification test, reported at the end of the column, is 546.995, confirming the validity of the estimations. The result in column (2) reveals a positive effect of instrumented *Log_L3_AQI* on health insurance purchases. To ensure robustness, we repeat our 2SLS estimation with the instrumental variable *L3_Inversion_Num*. Columns (3) and (4) show that the coefficients of *L3_Inversion_Num* and instrumented *Log_L3_AQI* are both statistically positive, consistent with expected.

However, it is worth noting that the 2SLS estimation may identify the local average treatment effect. For instance, thermal inversion primarily occurs during the winter. Identifying the average treatment effect within the winter subsample leads to a larger pollution coefficient compared to OLS estimation. It is also common in recent studies with same quasi-experimental methods [19, 21].

#### 4.2.3 The difference-in-difference regression based on the “2+26” plan

Our final identification strategy is difference-in-difference regression based on the “2+26” plan. Chinese government has promoted the “Air Pollution Prevention and Management Plan for the Beijing–Tianjin–Hebei region and its Surrounding Areas” for three consecutive years since 2017. The plan aims to improve the air quality for Beijing, Tianjin, and 26 other cities in the Beijing-Tianjin-Hebei area during the autumn and winter pollution episodes (October to March of the following year). The plan proposes “cure the winter disease in summer”, which means rectifying illegal pollutant discharge from industrial enterprises, promoting new energy, and taking other measures every summer (April to September) to reduce air pollution in winter pollution episodes. The “2+26” plan only reduces regional air pollution and has no direct impact on health insurance.

To better identify the casual effect of air pollution on the demand for health insurance, we estimate the following DID regression:

$$Y_{it}\text{=}\beta_{1}Treat\times Post_{it}+\beta_{2}Post_{t}+\beta_{3}X_{it}+D_{it}+\varepsilon_{it}$$ (4)

where *Y*_*it*_ refers to either air pollution or the demand for health insurance of city *i* on date *t*. *Treat*_*i*_ is a dummy variable that equals one for Beijing, Tianjin, Shijiazhuang, and Jinan, which are the cities covered by the “2+26” plan, and zero otherwise. *Post*_*t*_ is a dummy variable that equals one for the period after October 1, 2017, and zero for earlier. *Treat×Post*_*it*_ is the interaction term of *Treat*_*i*_ and *Post*_*t*_. The vector *X*_*it*_ and *D*_*it*_ denote the same weather controls and fixed effects as the model (1). The *Treat* variable is omitted due to collinearity with fixed effects.

**Fig 1:**
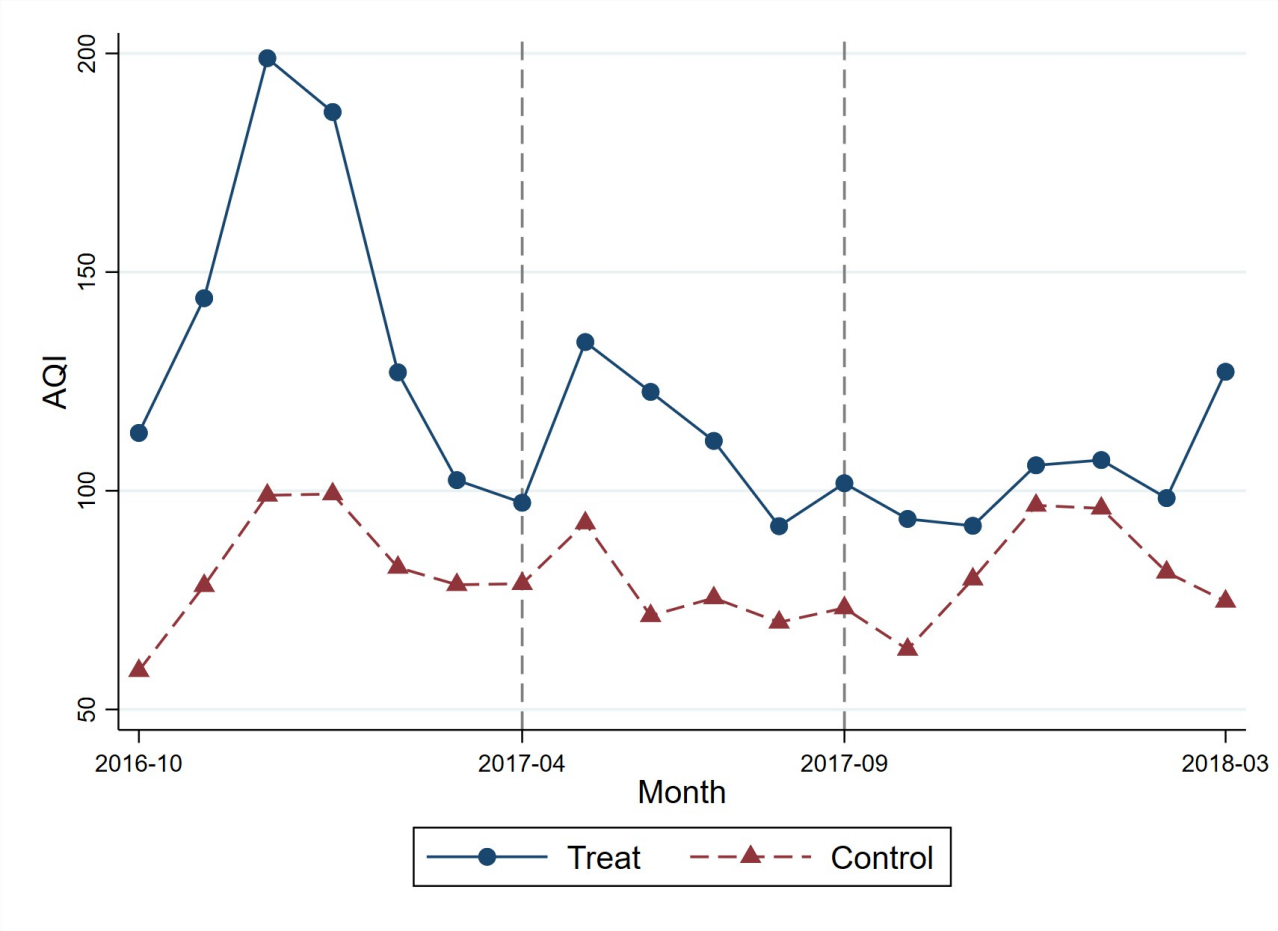
Monthly average AQI in treatment and control cities. Note: Fig 1 presents the monthly average AQI of treatment and control cities from October 2016 to March 2018. The solid dot line is treatment cities, including Beijing, Tianjin, Shijiazhuang, and Jinan, which are the cities covered by the "2+26" plan, and the dashed triangle line is control cities. The period from October 2016 to March 2017 is a pollution episode before the plan and from October 2017 to March 2018 is a pollution episode after the plan. The period from April to September 2017 is the first pollution prevention and management period.

At first, we intuitively evaluate the mitigation effect of the “2+26” plan. Our sample period covers a pollution episode from October 2016 to March 2017 before the plan and a pollution episode from October 2017 to March 2018 after the plan. Fig 1 shows the monthly average AQI of treatment and control cities from October 2016 to March 2018, where April to September 2017 was the first pollution prevention and management period. As we can see, the average AQI in treatment cities reduced significantly in October 2017 to March 2018 compared with the same period before the plan in October 2016 to March 2017, while control cities remained stable.

The results of DID regression are reported in Table 5. The dependent variable is *Log_L3_AQI* for column (1), while *Log_Num_contract* for columns (2) and (3). The coefficients on Treat*Post are significantly negative, implying that air quality improves and the demand for health insurance decreases in treatment cities. The “Three-year Action Plan to Win the Blue Sky Defense War (BSDW)” was enacted on June 27, 2018. For column (3), we exclude the sample period after the BSDW since it also covers the “2+26” cities. The coefficient of *Treat×Post* is still significantly negative at the 1% level. The DID results support the causal effect of air pollution on health insurance purchases.

**Table 5:** The “2+26” plan and health insurance purchases.

	AQI	Health Insurance	
Variables	(1)	(2)	(3)
*Treat×Post*	-0.2162***	-0.2185***	-0.1948**
	(-4.236)	(-2.653)	(-2.415)
*Post*	0.0008	0.1146***	0.0623
	(0.037)	(3.060)	(1.587)
Weather Controls	Yes	Yes	Yes
Fixed Effects	Yes	Yes	Yes
Adjusted *R*^*2*^	0.5148	0.7147	0.7129
N	34,656	34,656	31,084

Note: All regressions control for year-by-city, month-of-year-by-city, day-of-week, and holiday fixed effects, as well as a series of quadratic weather variables. The t-statistics are reported in parentheses based on standard errors clustered at the city-year level. Significance is indicated by

*** 1%

** 5%

* 10%.

To better understand the interpretation of DID results, we next examine the dynamic effects. We do this by including a series of indicators for each year before the “2+26” plan in model (5).

$$\begin{array}{c} Y_{it}\text{=}\eta_{1}Treat*Pre_{\text{it}}^{2014}+\eta_{2}Treat*Pre_{\text{it}}^{2015}+\eta_{3}Treat*Pre_{\text{it}}^{2016}+\eta_{4}Treat*Post_{it}\\ +\eta_{5}Pre_{\text{t}}^{2014}+\eta_{6}Pre_{\text{t}}^{2015}+\eta_{7}Pre_{\text{t}}^{2016}+\eta_{8}Post_{t}+\eta_{9}X_{it}+D_{it}+\varepsilon_{it} \end{array}$$ (5)

where *Pre*^*j*^_*t*_ equals one for the period after October 1, year *j*, and zero for earlier. *Treat×Post*^*j*^_*it*_ is the interaction term of *Treat*_*i*_ and *Pre*^*j*^_*t*_. The control variables and fixed effects are consistent with model (1). And the *Treat* variable is absorbed by fixed effects.

**Fig 2:**
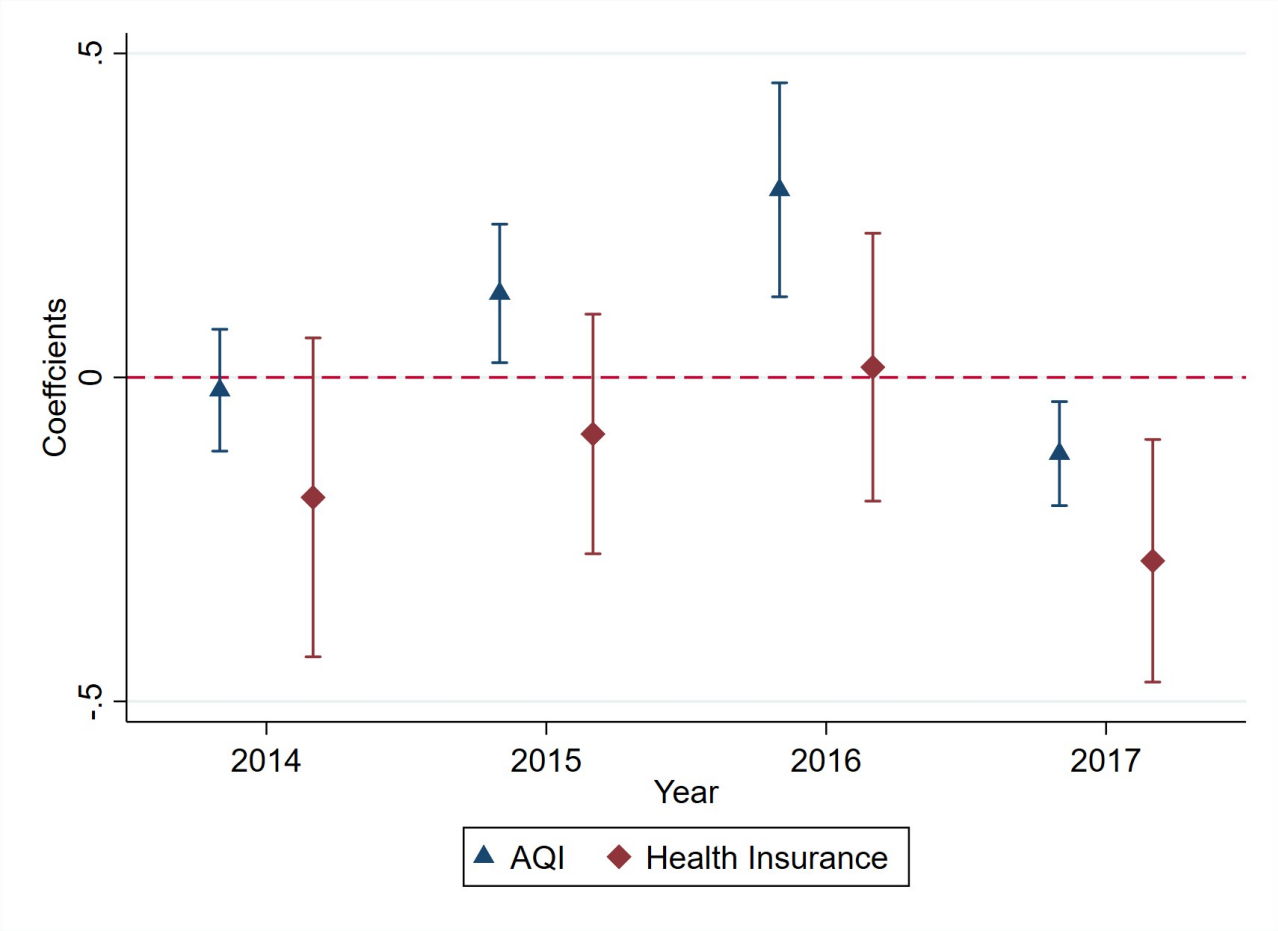
Parallel trends and dynamic effects. Note: Fig 2 reports the coefficient value and 95% confidence interval of each interaction term. The dependent variable of the dashed triangle line is *Log_L3_AQI*, and the dashed rhombus line is *Log_Num_contract*. The figure is constructed by re-estimating the DID model including the indicators for each year before the “2+26” plan.

Fig 2 presents the results. The coefficient trend on health insurance purchases indicates that there is no significant difference between treatment and control cities before. However, it significantly decreases to negative since the implementation of the “2+26” plan, which is consistent with the trend on air pollution. In total, Figs 1 and 2 provide supportive evidence that the parallel trends assumption is upheld.

### 4.3 Robustness checks

#### 4.3.1 Different windows for air pollution

In the baseline results, we use the average AQI over a three-day window to address the potential lagging effects. We also examine the two-day, four-day, five-day and six-day windows and replace the independent variable with the natural logarithm of the average AQI over different windows. The re-estimated results of model (1) are reported in Table 6. All coefficients on independent variable remain positive and statistically significant, indicating that our findings hold across different windows.

**Table 6:** Robustness checks using different windows for air pollution.

	Health Insurance			
	two-day	four-day	five-day	six-day
Variables	(1)	(2)	(3)	(4)
*Log_DW_AQI*	0.0358***	0.0381***	0.0369**	0.0326*
	(2.860)	(2.646)	(2.303)	(1.817)
Weather Controls	Yes	Yes	Yes	Yes
Fixed Effects	Yes	Yes	Yes	Yes
Adjusted *R*^*2*^	0.7144	0.7145	0.7144	0.7143
N	34675	34637	34618	34599

Note: All regressions control for year-by-city, month-of-year-by-city, day-of-week, and holiday fixed effects, as well as a series of quadratic weather variables. The t-statistics are reported in parentheses based on standard errors clustered at the city-year level. Significance is indicated by

*** 1%

** 5%

* 10%.

#### 4.3.2 Alternative measurements for air pollution

In the paper, we use the AQI, disclosed by the Chinese government, to measure air pollution. To further eliminate the potential interference of data manipulation on our results, we follow Jiang et al. (2022) [37] by employing PM2.5 density information from the Global Annual PM2.5 Grids database [38], as an alternative measure of air pollution. We replace the independent variable with the natural logarithm of the average PM2.5 in the city over a three-day window. The results are reported in Table 7. The coefficient of *Log_L3_PM2*.*5* is 0.0315 and is significantly positive, indicating that a 10% increase in the PM2.5 index leads to a 0.31% increase in health insurance sales in the city.

**Table 7:** Robustness checks using alternative measurements for air pollution.

	Health Insurance
Variables	(1)
*Log_L3_PM2*.*5*	0.0315***
	(3.087)
Weather Controls	Yes
Fixed Effects	Yes
Adjusted *R*^*2*^	0.7145
N	34656

Note: All regressions control for year-by-city, month-of-year-by-city, day-of-week, and holiday fixed effects, as well as a series of quadratic weather variables. The t-statistics are reported in parentheses based on standard errors clustered at the city-year level. Significance is indicated by

*** 1%

** 5%

* 10%.

### 4.4 Heterogeneity

Our results confirm that air pollution positively affects the demand for health insurance. However, the risk attitudes of individuals vary widely based on factors such as age, gender, and family background [39]. According to The Report of 2018 China Commercial Health Insurance Development Index, there are significant differences in perceptions of health insurance and awareness of health management among groups with different characteristics. Therefore, we conduct further analysis to explore the heterogeneous response of different groups based on their characteristics. Firstly, we categorize the consumers into groups of female and male by gender and into groups of younger (40 and under) and older (above 40) by age. Next, we aggregate the daily number of health insurance contracts to each group in each city. Finally, we replace the dependent variable with the natural logarithm of the number of health insurance contracts of each city-group-date.

Table 8 shows the responses to air pollution of different groups. The coefficients on *Log_L3_AQI* are only significant for the female and younger, whereas the effect of air pollution on health insurance purchases is slight and insignificant for the male and older. The results suggest that female and younger groups are more sensitive and responsive to air pollution.

**Table 8:** Heterogeneous effects of air pollution on health insurance purchases.

	Health Insurance			
	female	male	40 and under	above 40
Variables	(1)	(2)	(3)	(4)
*Log_L3_AQI*	0.0417***	0.0150	0.0401***	0.0126
	(3.542)	(1.382)	(3.193)	(1.308)
Weather Controls	Yes	Yes	Yes	Yes
Fixed Effects	Yes	Yes	Yes	Yes
Adjusted *R*^*2*^	0.6858	0.6382	0.7129	0.5094
N	34,656	34,656	34,656	34,656

Note: All regressions control for year-by-city, month-of-year-by-city, day-of-week, and holiday fixed effects, as well as a series of quadratic weather variables. The t-statistics are reported in parentheses based on standard errors clustered at the city-year level. Significance is indicated by

*** 1%

** 5%

* 10%.

## 5. Mechanisms

### 5.1 Health management

Air pollution poses a threat not to be overlooked to human health and life expectancy, as it can increase the incidence of morbidity and mortality for cardiorespiratory diseases [3, 40]. Consequently, health management is possibly the most straightforward explanation for the effect of air pollution on health insurance purchases. Health insurance is recognized as an essential tool for managing health risks and improving healthcare [41]. The development level of local commercial health insurance reflects the awareness of health management for residents. To gauge this, we use local commercial health insurance density and penetration in the previous year as proxy variables for awareness of health management. The density of health insurance is determined by the ratio of local health insurance premiums to the permanent population. Health insurance penetration is evaluated by the percentage of local health insurance premiums in relation to the GDP.

We re-estimate the model (1) with the subsample grouped by the median of health insurance density. The results are reported in columns (1) and (2) of Table 9. The coefficient on *Log_L3_AQI* is 0.0687 and significant at the 1% level in the high health insurance density subsample, whereas it is 0.0184 and statistically insignificant in the low health insurance subsample. The difference in their coefficients is also significant at the 1% level. Columns (3) and (4) of Table 9 are the regression results for groups divided by the median of health insurance penetration. Although the coefficients of *Log_L3_AQI* are both significant, the difference between them is still significant positive at the 5% level. Overall, the insurance effect of air pollution is significantly larger in the subsample with better health management awareness. This evidence suggests that awareness of health management drives consumers to purchase insurance when exposed to air pollution.

**Table 9:** Health management and health insurance purchases.

	Health Insurance			
	high density	low density	high penetration	low penetration
Variables	(1)	(2)	(3)	(4)
*Log_L3_AQI*	0.0687***	0.0184	0.0479**	0.0300*
	(3.579)	(1.042)	(2.408)	(1.697)
	diff (1)-(2)	0.0503***	diff (3)-(4)	0.0179**
Weather Controls	Yes	Yes	Yes	Yes
Fixed Effects	Yes	Yes	Yes	Yes
Adjusted *R*^*2*^	0.7492	0.5543	0.7332	0.6685
N	17,163	17,493	17,161	17,495

Note: All regressions control for year-by-city, month-of-year-by-city, day-of-week, and holiday fixed effects, as well as a series of quadratic weather variables. The t-statistics are reported in parentheses based on standard errors clustered at the city-year level. Significance is indicated by

*** 1%

** 5%

* 10%.

### 5.2 Social interaction

**Fig 3:**
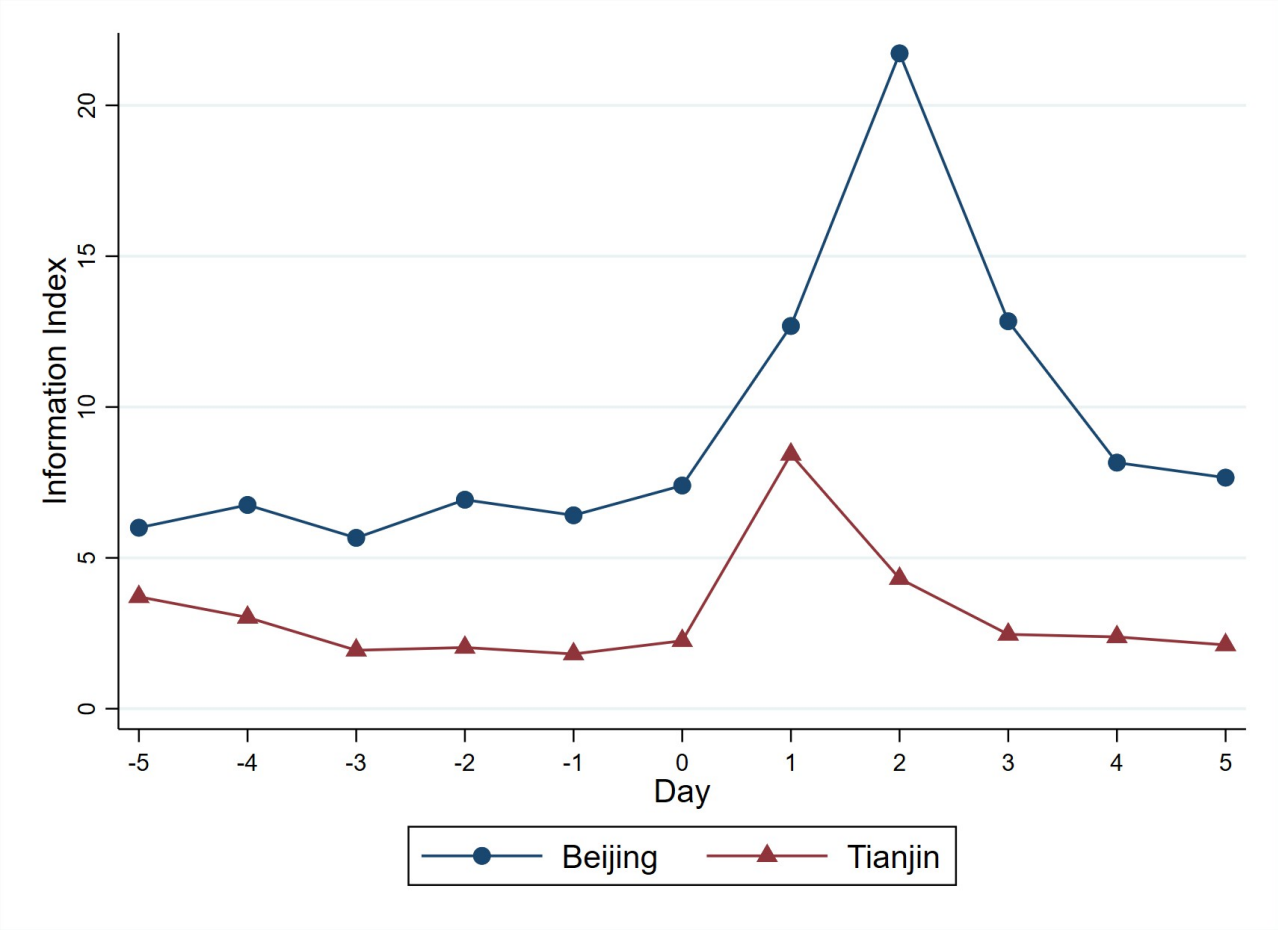
Information index on “air pollution” in Beijing and Tianjin. Note: Fig 3 shows the Baidu information index on “air pollution” for five days before and after the pollution days in Beijing and Tianjin. The y-axis is the information index scaled by 10000. The x-axis is five days before and after the pollution day (Day 0). The day when the increase of AQI exceeds the standard deviation of the AQI for the past year in the city is considered as a pollution day (Day 0).

Air pollution significantly increase the online search for anti-PM2.5 masks, air filters, and migration [32, 42]. From the perspective of daily life, both severe air pollution events and defensive behaviors provide topics for social interaction. Although it is hard for us to measure social interaction in the context of air pollution, we use the Baidu information index on “air pollution” to simply assess its influence on online social interaction. The Baidu information index is calculated based on the number of local netizens’ commenting, reposting, likes, and dislikes on related topics since July 1, 2018, which can be used as a direct measure of online social interaction. Fig 3 shows the information index on “air pollution” for five days before and after the pollution day in Beijing and Tianjin, which are more polluted cities in our sample. A pollution day (Day 0) as a day on which the increase of AQI exceeds the standard deviation of the AQI for the past year in the city [43]. We find that the information index on “air pollution” is notably higher after the pollution days and generally returns to the normal three or four days after. Further, we use the natural logarithm of the information index as the dependent variable and the result is reported in column (1) of Table 10. The significant positive coefficient on *Log_L3_AQI* indicates that air pollution promotes social interaction, as expected.

**Table 10:** Social interaction and health insurance purchases.

		Health Insurance			
	Index	high commu_prop	low commu_prop	high commu_chfs	low commu_chfs
Variables	(1)	(2)	(3)	(4)	(5)
Log_L3_AQI	0.3981***	0.0687***	0.0090	0.0574***	0.0194
	(4.788)	(3.314)	(0.599)	(2.788)	(1.039)
		diff (2)-(3)	0.0597***	diff (4)-(5)	0.0380***
Weather Controls	Yes	Yes	Yes	Yes	Yes
Fixed Effects	Yes	Yes	Yes	Yes	Yes
Adjusted R^2^	0.5802	0.6401	0.7676	0.6723	0.7275
N	3,496	17,507	17,149	15,322	15,686

Note: All regressions control for year-by-city, month-of-year-by-city, day-of-week, and holiday fixed effects, as well as a series of quadratic weather variables. The t-statistics are reported in parentheses based on standard errors clustered at the city-year level. Significance is indicated by

*** 1%

** 5%

* 10%.

Social interaction can help people to get open but less salient public information due to the information transfer in interactive scenarios [22]. In addition, social interaction may update their beliefs about the hazards of air pollution and the role of health insurance considering the effect of herd mentality on people’s decision-making [44, 45]. Hu (2022) [22] also provides evidence that social interaction significantly influences households’ decisions regarding flood insurance in the US. To measure social interaction, we follow Shi et al. (2015) [46] and use the expenses on transportation and communication as a proportion of total household consumption (*commu_prop*), which are from the China cities’ statistical yearbooks.

An additional factor to consider is the household spending on communication from the China Household Finance Survey (CHFS) in 2013, 2015, 2017, and 2019, which is conducted by the Southwestern University of Finance and Economics (*commu_chfs*)^3^. The variable is derived from households and then adjusted for sampling weights at the city level. In years without a survey, the missing data is supplemented with the average of the preceding and succeeding years.

Columns (2) to (5) of Table 10 report the results of subsamples categorized by the median of two social interaction indicators. According to columns (2) and (3), we find that the marginal effect of air pollution is 0.687% and significant at the 1% level in the subsample with a high level of social interaction, while it is 0.090% and statistically insignificant in another subsample. Similar results are observed in columns (4) and (5). The coefficient of *Log_L3_AQI* is positively significant only in the subsample with a high level of social interaction. In summary, the results indicate that social interaction significantly increases health insurance purchases among individuals exposed to air pollution.

## 6. Marginal willingness to pay for air pollution

Although it is evident that air pollution substantially impacts on the number of health insurance purchases, its economic magnitude cannot be directly predictive. According to insurance transaction data, we know that consumer *j* in the city *i* purchase a health insurance contract at price *P* to defend against the pollution level *L3_AQI*. We empirically construct the following model to roughly estimate the defensive marginal willingness to pay (MWTP) for air pollution using individual-level transaction data:

$$L\text{og\_}MkShare_{ijt}=\delta L3\_AQI_{ijt}+\gamma P_{ijt}+\eta X_{it}+D_{it}+I_{j}+\varepsilon_{ijt}$$ (6)

where *MkShare*_*ijt*_ is the market share of city *i* on date *t* when consumer *j* purchases health insurance, which is the ratio of the number of health insurance contracts sold to the permanent residents of that year considered as potential health insurance buyers [23]. *L3_AQI*_*ijt*_ is the average AQI of the city *i* over a three-day window faced by consumer *j*. *P*_*ijt*_ is the yearly premium of health insurance sold to consumer *j*. *X*_*it*_ is weather control variables, and *D*_*it*_ denotes fixed effects defined as the model (1). *I*_*j*_ represents consumer fixed effects, controlling for the characteristics of consumers. Standard errors are clustered at the city-year level.

In model (6), *δ* represents the marginal effect of air pollution, and *γ* represents the marginal effect of price. The marginal willingness to pay (MWTP) for one unit air pollution increase can be estimated by *-δ/γ*, which is the marginal rate of substitution between air pollution and price. Table 11 reports the results. Column (1) is estimated without any control variables. Column (2) is our main specification model, including weather control variables. As shown in the last row of Table 11, the defensive marginal willingness to pay for a one-unit increase in AQI is 302.23 Chinese Yuan per year per person. Given the data available, the estimated defensive cost of air pollution, conditional on health insurance purchases, is approximately 1.70% of consumer income^4^. Although estimates with different pollutants cannot be directly compared, our estimate is mostly similar with Gao et al. (2023) [24], who propose that a median-income Chinese citizen is willing to pay 336 Chinese Yuan, accounting for approximately 1.69% of the income, to migrate for a 1 *μg/m*^*3*^ reduction in PM2.5 concentration after real-time pollution information is available.

**Table 11:** The marginal willingness to pay for air pollution.

	Log (Market Share)	
Variables	(1)	(2)
L3_AQI (δ)	1.4375***	0.8848***
	(4.270)	(2.725)
P (γ)	-0.0029***	-0.0029***
	(-2.735)	(-2.811)
Weather Controls	No	Yes
Fixed Effects	Yes	Yes
Adjusted R^2^	0.8646	0.8670
N	213,535	213,535
MWTP (-δ/γ)	487.3423*	302.2282*

Note: All regressions control for year-by-city, month-of-year-by-city, day-of-week, holiday, and the consumer fixed effects, as well as a series of quadratic weather variables. The t-statistics are reported in parentheses based on standard errors clustered at the city-year level. Significance is indicated by

*** 1%

** 5%

* 10%.

## 7. Conclusion

The research empirically tests the impact of air pollution on defensive behavior using unique transaction-level data from nineteen cities in China from 2014 to 2018. We find that health insurance purchases are positively affected by air pollution. We also observe that the defensive behavior of air pollution is non-linear and pronounced when the AQI exceeds 200. Moreover, we evaluate the defensive cost by estimating the marginal willingness to pay for air pollution.

Importantly, we also examine the causal effect of air pollution on defensive behavior using three identification strategies: (1) a placebo test involving the non-health insurance, consisting of accident and endowment insurance, sold by the same company; (2) ventilation coefficient and thermal inversion as instrumental variables for air pollution; and (3) difference-in-difference analysis based on the “2+26” plan. All empirical strategies confirm that air pollution has a positive effect on health insurance.

We explore the mechanisms underlying the purchase effect associated with air pollution. We find that the marginal effect of air pollution is significantly larger in the subsamples with high awareness of health management and high level of social interaction. In conclusion, the results suggest that health management and social interaction drive consumers to purchase insurance when exposed to air pollution.

### Footnotes

^1^ The product is M2T1NXFLX version 5.12.4.

^2^ The product is M2I6NPANA version 5.12.4.

^3^ Despite the extensive and representative sample in CHFS, survey data for Dongguan and Wuxi is still unavailable.

^4^ The average income for consumers in our sample is 17824.68 Chinese Yuan.

## Supporting information

S1 Appendix (DOCX)

S1 Dataset (DTA)
