# Supplementary material for: Air pollution and defensive behavior: Evidence from transaction data in China
Source: PLoS One. 2024 Nov 7;19(11):e0307295. doi: 10.1371/journal.pone.0307295 (PMC11542840; doi:10.1371/journal.pone.0307295)
Supplement: S1 Appendix — (DOCX) [file pone.0307295.s001.docx]

**S1 Appendix**

**Table S1. Definitions of variables.**

| **Variables** | **Definitions** |
| --- | --- |
| *Num_Contract* | the daily number of health insurance contracts sold by the company in the city. |
| *Log_Num_Contract* | the natural logarithm of the daily number of health insurance contracts sold by the company in the city. |
| *L3_AQI* | the average AQI of the city over a three-day window. |
| *Log_L3_AQI* | the natural logarithm of the average AQI of the city over a three-day window. |
| *I (AQI ≤100)* | equals one when the average AQI is less than or equal to 100, and zero otherwise. |
| *I (100 < AQI ≤150)* | equals one when the average AQI is greater than 100 and less than or equal to 150, and zero otherwise. |
| *I (150 < AQI ≤200)* | equals one when the average AQI is greater than 150 and less than or equal to 200, and zero otherwise. |
| *I (200 < AQI ≤300)* | equals one when the average AQI is greater than 200 and less than or equal to 300, and zero otherwise. |
| *I (AQI >300)* | equals one when the average AQI is greater than 300, and zero otherwise. |
| *Log_ AQI* | the natural logarithm of the average AQI of the city in the date. |
| *Log_DW_AQI* | the natural logarithm of the average AQI of the city over different windows. |
| *Log_L3_PM2.5* | the natural logarithm of the average PM2.5 of the city over a three-day window. |
| *Temperature* | the average temperature of the city in the date (*℃*). |
| *Temperature^2^* | the quadratic of the daily average temperature. |
| *Precipitation* | the average precipitation of the city in the date (*mm*). |
| *Precipitation^2^* | the quadratic of the daily average precipitation. |
| *Humidity* | the average humidity of the city in the date (*%*). |
| *Humidity^2^* | the quadratic of the daily average humidity. |
| *Wind_speed* | the average wind speed of the city in the date (*m/s*). |
| *Wind_speed^2^* | the quadratic of the daily average wind speed. |
| *L3_VC* | the average ventilation coefficient of the city over a three-day window. |
| *Log_L3_VC* | the natural logarithm of the average ventilation coefficient of the city over a three-day window. |
| *L3_Inversion_Num* | the number of thermal inversions occurring in the city over a three-day window. |
| *Treat* | equals one for the cities covered by the “2+26” plan, and zero otherwise. |
| *Post* | equals one for the period after the “2+26” plan, and zero otherwise. |
| *density* | the ratio of health insurance premiums to the permanent population in the city. |
| *penetration* | the percentage of health insurance premiums to GDP in the city. |
| *commu_prop* | the proportion of transportation and communication of total household consumption in the city. |
| *commu_chfs* | the household average spending on communication in the city calculated from CHFS. |
| *MkShare* | the ratio of the number of health insurance contracts sold to the permanent residents in the city. |
